# Linking attractive interactions and confinement to the rheological response of suspended particles close to jamming

**DOI:** 10.1007/s10035-017-0770-1

**Published:** 2017-11-11

**Authors:** Michael A. Jones, Christopher Ness

**Affiliations:** 1 Cavendish Laboratory, University of Cambridge, Cambridge, CB3 0HE UK; 2 Department of Chemical Engineering and Biotechnology, University of Cambridge, Cambridge, CB3 0AS UK

**Keywords:** Suspension, Rheology, Attraction, Confinement

## Abstract

We study the response to simple shear start-up of an overdamped, athermal assembly of particles with tuneable attractive interactions. We focus on volume fractions close to the jamming point, where such systems can become disordered elastoplastic solids. By systematically varying the strength of the particle–particle attraction and the volume fraction, we demonstrate how cohesion and confinement individually contribute to the shear modulus and yield strain of the material. The results provide evidence for the influence of binding agents on the rheology of dense, athermal suspensions and describe a set of handles with which the macroscopic properties of such materials can be engineered.

## Introduction

Yield-stress fluids have a vast range of uses in everyday life and in industry, from cement and ceramic pastes to shaving gel and mayonnaise [1, 2]. Their microscopic structure enables them to flow plastically if they are submitted to a stress above some critical value, otherwise they deform in a finite way, resembling an elastic, structurally disordered solid. This behaviour makes them ideal materials for a host of applications, and consequently they have long been the subject of extensive research [3–7]. There is an ongoing effort to further enhance the rheological properties of such materials, focussing on the specificity with which attractive interactions might be tailored to tune the yield stress behaviour. Recent experimental developments have made possible materials with particle–particle attractions that may be precisely tuned, and selectively ‘switched’ on and off using external stimuli [8–10]. This can provide an extra handle with which the macroscopic properties of dense suspensions can be controlled. Particles functionalised with single-stranded DNA, for example, can be designed to bind only with particles coated with the complementary strand, and if the surface functionalisation can be added in such a way as to make it mobile on the particle surface [11] then very complex and selective interactions can be realised. Predicting the stress response of such designer materials under shearing, with the goal of designing and creating the ideal material for given applications remains an exciting challenge to soft matter physicists and engineers.

At the same time, the emergence of yield-stress behaviour during the transportation and storage of nominally dry granular materials remains a related engineering challenge across manufacturing and processing industries [12, 13]. In such scenarios, attractive interactions may arise between particles as a result of electrostatic interactions or liquid bridging due to environmental moisture, for example. The material may then be described at a mesoscopic level as a very dense assembly of sticky particles, which are themselves plastically deformable [14]. Although various studies have explored the role of particle adhesion at this mesoscopic level with respect to specific industrial operations including powder processing and tablet formation, a thorough understanding of the influence of cohesion in dense grains under simple shear remains elusive.

Though seemingly distinct at an application level, the fundamental challenge of understanding yield stress behaviour in designer soft solids and cohesive grains shares many parallels, not least the need to reconcile the competing roles of attraction and confinement at high volume fractions. This challenge is reflected in the recent literature: suspensions of repulsive particles have been the subject of a considerable amount of research over recent years, but attention is increasingly turning to the role of attractive interactions in determining the rheology of such fluids [15–19]. Indeed, the phase diagram for attractive *dry* granular materials has been revealed recently by Refs. [6, 20], who identified adhesive close and loose packing volume fractions, analogous to their repulsive-particle counterparts. That jamming can be achieved at such low volume fractions naturally reflects the link between adhesive non-Brownian systems and colloidal gelation. While there is mounting evidence of the role that hydrodynamics can play in gelation [21], it remains unclear whether similar effects are relevant to saturated wet granular systems.

In the present article, discrete element simulations are used to model disordered assemblies of suspended particles very close to the point of marginal rigidity, which may be reached either through increased particle–particle connectivity or through increased confinement. These simulations account for differing particle–particle attraction strength and varying volume fractions, and we study the mechanical response to simple shear across the transition at which the system becomes solid. Distinguishing between solid and liquid phases in attractive systems is in general nontrivial, and a complete rheological characterisation requires a consideration of the complex moduli as functions of the frequency and amplitude of the applied strain. For simplicity, we define a flowing region in which the start-up stress is roughly linear in the strain rate, and a jammed region where the start-up stress is roughly linear in the strain magnitude. We do not, though, discuss in detail the non-Newtonian nature of each of these regimes.

Our systematic approach provides a guide for understanding the rheological consequences of emergent yield stresses during industrial processing, and tuning the yielding behaviour of soft materials more generally.

## Methods

In each simulation we consider a three dimensional, periodic system of 5400 spheres, with the dimensions of the enclosing box being varied to achieve desired volume fractions $\phi$. To avoid crystallisation, each system contains a 1:1 mixture of particles with diameters *d* and 1.4*d*. The particles are subjected to simple shear flow with strain $\gamma$ at fixed rate $\dot{\gamma }$, with the flow direction in *x* and the gradient in *y*. Operating at fixed shear rate allows us to prevent shear banding, which might otherwise arise in such attractive particle suspensions [22]. We compute the Newtonian dynamics for all particles at each timestep, with particles being subjected to hydrodynamic and contact forces.

For particle pair $\alpha$ (consisting of particles *i* and *j* with diameters $d_i$ and $d_j$) with centre-to-centre vector ${\varvec{r}}_\alpha$, relative normal velocity (along ${\varvec{r}}_\alpha$) ${\Delta }\mathbf {v}_\alpha$, separated by surface-to-surface gap $h = |{\varvec{r}}_\alpha | -\frac{1}{2} (d_i + d_j)$, the hydrodynamic force $\mathbf {F}^h_\alpha$ is computed according to

1 $$\begin{aligned} \mathbf {F}^{h}_{\alpha } = \frac{3\pi \eta _{f} d^2_{\alpha }}{2} \frac{1}{h_{\mathrm{eff}}}{\Delta }\mathbf {v}_{\alpha }\text {,} \end{aligned}$$

where $\eta _{f}$ is the viscosity of the background fluid, $d_\alpha = d_id_j/(d_i+d_j)$ and $h_{\mathrm{eff}}$ is given by:

2 $$\begin{aligned} h_\text {eff} = \left\{ \begin{array}{ll} h &{} \quad \text {for } 0.001d\le h\le 0.05d\\ 0.001d &{} \quad \text {for h<0.001d} \end{array} \right. \end{aligned}$$

where *d* is the diameter of the smaller particle. We choose 0.001*d* as an asperity length scale at which the lubrication forces cease to diverge. Hydrodynamic forces are set to zero when $h>0.05d$.

Individual particles *i* are further subjected to a Stokes drag force $\mathbf {F}^h_i = 3\pi \eta _{f} d_i (\mathbf {v}_i - y_i\dot{\gamma })$ due to their relative velocity with the background streaming flow. The contact force $\mathbf {F}^c_\beta$ represents a nonhydrodynamic interaction experienced by two particles $\beta$ when they come into contact with one another, which occurs when the distance between the particle centres is less than $d_{\beta }$ (which is given by $0.5(d_i+d_j)$). For a particle pair with centre-to-centre vector ${\varvec{r}}_\beta$, we define the overlap as $\delta = d_\beta -|{\varvec{r}}_\beta |$. A contact occurs wherever $\delta >0$, and we quantify the average number of such contacts per particle as $\langle Z \rangle$. The interaction force is then simply given by

3 $$\begin{aligned} \mathbf {F}^c_{\beta } = k\delta \mathbf {n}_{\beta } - F_{A}\mathbf {n}_\beta \text {,} \end{aligned}$$

where $\mathbf {n}_\beta$ represents the centre-to-centre unit vector and is equivalent to ${\varvec{r}}_\beta /|{\varvec{r}}_\beta |$, and *k* is the constant stiffness, which we set to 10,000 [units of force per unit distance]. The second term represents subtraction of a constant normal force with magnitude $F_{A}$, which has the effect of introducing a simple, short ranged, isotropic attraction between the particles. Not wishing to tie ourselves to a specific form of attractive potential [23], we demonstrate that this simplistic form is sufficient to obtain the desired crossover from liquid-like to solid-like properties. This approach paves the way for more complex interactions to be modelled between the particles in ongoing works, for example frictional forces, elastoplastic potentials or anisotropic attractive potentials. Particle trajectories are evolved with time according to the above forces using a Velocity–Verlet scheme implemented in LAMMPS [24].

For the purposes of these simulations, the dimensional parameters and their units are particle diameter *d* [length], fluid viscosity $\eta _{f}$ $[\hbox {mass}/(\hbox {length}\times \hbox {time})]$, particle stiffness $\textit{k}$ [mass/time$^{2}$], particle and fluid density $\rho$ [mass/length$^3$] and shear rate $\dot{\gamma }$ [1/time]. We express $F_{A}$ in units of *kd* throughout. Particle inertia is mitigated throughout by setting $\rho \dot{\gamma }d^2/\eta _{f}\sim 10^{-2}$, and we set $\dot{\gamma }d/\sqrt{k/\rho d}\sim 10^{-4}$.

We further calculate the stress tensor of the system, accounting for contributions of the different forces acting between the particles according to

4 $$\begin{aligned} {\varvec{\sigma }} = \frac{1}{V} \left[ \sum _{\alpha =1}^{N^h} \mathbf {r}_\alpha \otimes \mathbf {F}_\alpha ^h + \sum _{\beta =1}^{N^c} \mathbf {r}_\beta \otimes \mathbf {F}_\beta ^c \right] , \end{aligned}$$

where *V* is the volume of the system, $\mathbf {r}_\alpha$ and $\mathbf {r}_\beta$ represent vectors pointing from centre-to-centre of hydrodynamically interacting and contacting particles respectively, and $N^h$ and $N^c$ represent the total number of hydrodynamically interacting and contacting pairs, respectively. From the stress tensor $\varvec{\sigma }$ the component corresponding to shear stress, that in the $\textit{xy}$ plane, is identified and referred to as $\sigma$ for simplicity. The stress values quoted herein have dimensions of $\textit{k/d}$. For the cases where a suspension viscosity is given, this was calculated by dividing the stress response $\sigma$ at large values of shear strain (after the system has yielded and the stress has reached its large strain value) by the shear rate $\dot{\gamma }$. The viscosities are quoted as relative viscosities, $(\sigma /\dot{\gamma })/\eta _{f} = \eta /\eta _{f}$.

## Results

**Fig. 1:**
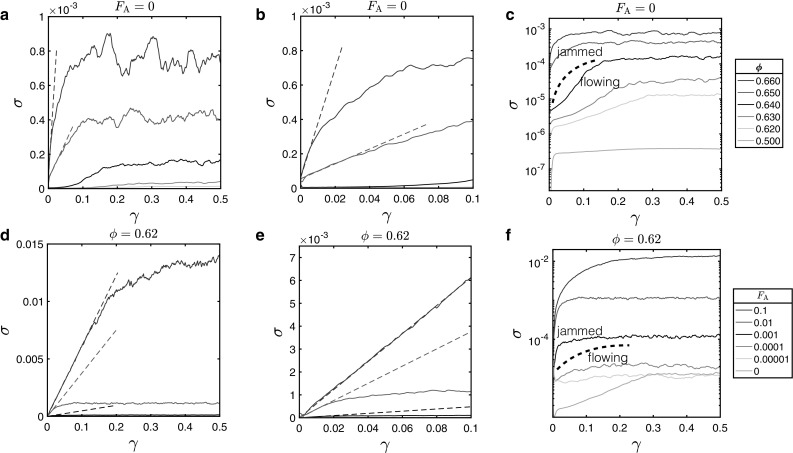
Stress–strain curves for simulations of a series of particle suspensions. **a**–**c** Zero attractive force ${F}_{\text {A}} = 0$, and the volume fraction ${\phi }$ is varied. **d**–**f** Fixed volume fraction ${\phi } = 0.620$ and the attractive force strength ${F}_{\text {A}}$ is varied. **a**, **d** Stress–strain curves on a linear scale over all strain values, while **b**, **e** show the linear plots at low strain. The dashed lines indicate the initial gradients (from which we obtain the elastic moduli, see Fig. 3) for those systems which are jammed to begin with and so deform elastically for small strains. **c**, **f** Data plotted against a logarithmic scale on the y-axis—the dotted lines indicate the boundaries between regions where the system has jammed and flowing behaviour. The legends show the volume fractions (**a**–**c**) and attractive forces (**d**–**f**) to which each line corresponds

Using the simulation results presented here we aim to illustrate how switching on a simple attractive interaction of a particular strength between the particles in a suspension can have an effect comparable to that of increasing the volume fraction by a certain amount: introducing, or increasing the magnitude of, the elastic response under shear. We thus look first at the macroscopic rheological response of the system to the application of external shear up to $\gamma =0.5$ by measuring the stress generated $\sigma$. From the stress–strain curves in Fig. 1 it can be seen that increasing either the volume fraction or attractive force produces a higher valued stress response in the system upon the application of shear. For the case where $\phi$ is increased, this can be understood in terms of the particles being forced to pack more closely together, thus increasing the confinement and making the system jam, resembling a solid. Increasing $\textit{F}_{\mathrm{A}}$ causes a solid-like response because of the resulting higher degree of connectivity through the suspension which results from percolating gel-like networks of contacts [25].

From these plots we can also identify the regions in which the behaviour of the system resembles that of a jammed solid, or is flowing. As discussed in the Introduction, we identify jammed states as those systems with an elastic response at small strain, i.e. that are linear in $\sigma$ versus $\gamma$, and flowing states as those that are more linear in the strain rate $\dot{\gamma }$. The boundary between the two is most clearly seen in (c) and (f), where the stress response has been plotted on a logarithmic axis. In this way we can approximate the minimum volume fraction (in the non-attractive case) or attractive strength (at fixed $\phi$) required to obtain a solid-like response at low shear strain. In the absence of attraction, the volume fraction required, $\phi _c$, is between 0.64 and 0.65, close to random close packing as expected, and for a volume fraction $\phi =0.62$ an ${F}_{\text {A}}$ of between 0.0001 and 0.001 *kd* is needed. For a glass sphere with Young’s Modulus $\sim 10^{10}$ Pa this represents the force required to deform the surface by $\sim 10^{-6}$ of the diameter. Comparing (c) and (f) we note that the incorporation of an attractive force ${F}_{\text {A}} = 0.01$ between particles for a system of volume fraction $\phi =0.62$, which would otherwise flow like a liquid, can result in a solid-like material which deforms with a shear modulus and yield stress comparable to that of a material with $\phi =0.66$ for ${F}_{\text {A}}=0$, which would be well within the jammed regime.

**Fig. 2:**
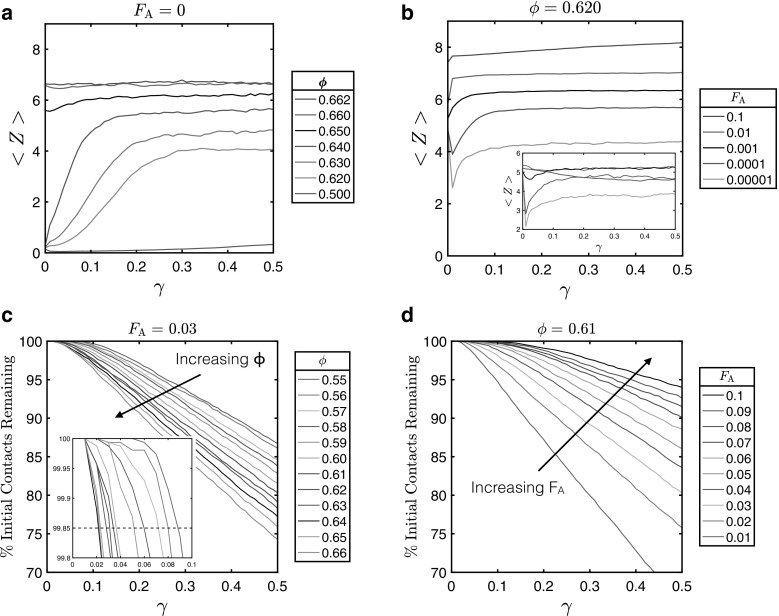
Coordination numbers across volume fractions and attractive strengths. **a**, **b** Average coordination numbers ${\langle Z \rangle }$ at each strain value for **a** a range of volume fractions ${\phi }$ (${F}_{\text {A}}=0$) and **b** a range of attractive forces ${F}_{\text {A}}$ (${\phi }=0.620$). Inset (**b**) shows the contact number defined at $\delta '>0$ for $\delta ' = (d_\beta -F_A/k)-|{\varvec{r}}_\beta |$; **c**, **d** show the percentage of initial contacts, those present at ${\gamma }=0$, which remain after each shear strain value in the simulation, for **c** constant ${F}_{\text {A}}$ and **d** constant ${\phi }$. Inset (**c**) shows the threshold remaining contacts (chosen to be 99.85%) below which the system is regarded as having yielded. We define $\gamma =\gamma _c$ at this point

To better understand the origin of this changing macroscopic behaviour it is important to consider the structure of these systems at the microscopic level. The average number of particle–particle contacts $\langle Z\rangle$ and the rate at which shearing destroys the initial microstructure are indicative of whether the system is jammed or flowing, and can locate the point at which jamming and yielding occur, respectively. For lower particle volume fractions the coordination number clearly increases as shear is applied and a shear-induced contact network is assembled, Fig. 2a. The detailed evolution of the microstructure in this region, as well as a characterisation of the corresponding stress response (which we presented in Fig. 1c) have been discussed in detail elsewhere [26]. For ${\phi }=0.650$ and above, however, the application of shear strain has little effect on the average coordination number. The denser, more elastic systems already have an established contact network spanning the whole system before any shearing takes place, meaning the coordination number is already close to its critical value of $\langle Z \rangle =6$, so little change is observed. This result is quantitatively consistent with constraint-counting arguments for isostaticity in disordered systems [27]. For those systems with a volume fraction below 0.650, where the average number of contacts is initially quite small, the network of contacts evolves as shear strain is applied. It is clear from Fig. 2b that the coordination number increases with attractive force, as particles are pulled towards one another. For the weaker interaction strengths, particles are less tightly bound and as such are separated slightly upon the onset of shear, hence the observed decrease in $\langle Z \rangle$, before being drawn towards each other once more.

For the attractive systems we further define a ‘repulsive’ contact number which gives the number of contacts that have $\delta '>0$ for $\delta ' =(d_\beta -F_A/k)-|{\varvec{r}}_\beta |$, Figure 2b (Inset). This quantity identifies those particles that are in the repulsive part of the overall interaction potential. For $F_A<0.1$, we find analogous behaviour to the standard definition of $\langle Z \rangle$, though with the numbers reduced. Interestingly, the systems identified as ‘jammed’ previously have, according to this new definition, contact numbers lower than the critical value of 6. This suggests that the system is stabilised not by repulsive interactions alone, but by a combination of repulsion and attraction. For $F_A=0.1$, we observe anomalous behaviour whereby the repulsive contact number is lower than for more weakly attractive systems. This is due to steric effects. In this case, particles must overlap by 10% of their diameter to reach the repulsive region. While this is permissible for isolated contacts, once a sphere has, say, 4 contacts at 10% overlap, it is more difficult to place a fifth or sixth sphere around the same central particle with 10% overlap because the radius of the central particle is effectively reduced by 10%.

We take a snapshot of the initial contact topology at $\gamma =0$ and count all those initial contacts that remain intact as shearing proceeds. To do this, we first construct a list of pairwise particle–particle contacts at time $t=0$, by performing a search of particles with centre-to-centre separation less than $d_\beta$. We then repeat this computation at all subsequent timesteps, each time counting all those contacts from $t=0$ that are still present. The result is presented in Fig. 2c, d. We can see from (d) that a larger attractive force between the particles causes, as expected, a greater percentage of contacts to remain over the duration of the simulation. Similarly, from (c), a lower volume fraction of particles with the same ${F}_{\text {A}}$ results in a higher number of initial contacts persisting, as the particles are better able to stick together due to fewer collisions with other particles, and increases adherence to the background streaming flow. We define the critical strain $\gamma _c$ at the point where the number of remaining contacts drops below 99.85%. This was found to be a convenient choice at which the predicted values of $\gamma _c$ closely correspond to the positions where the stress-strain curves begin to deviate from linearity for the jammed systems.

**Fig. 3:**
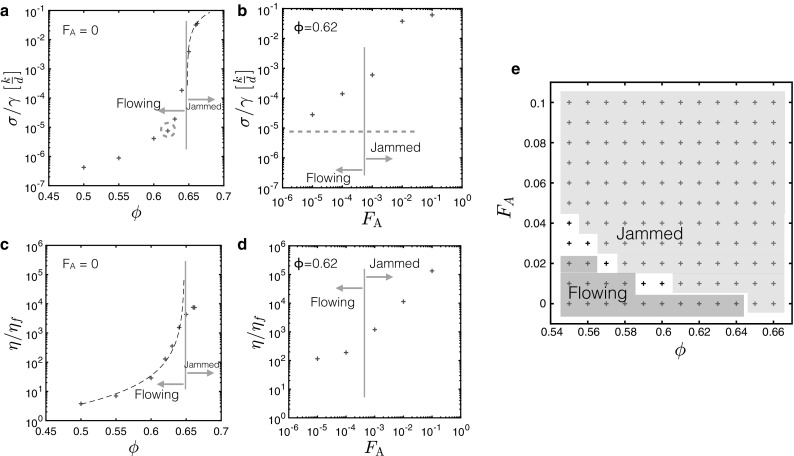
Transition from flowing to jammed response as functions of volume fraction and attractive strength. **a**, **b** Plots of the shear modulus (the gradient ${\sigma }$/${\gamma }$, taken over low values of strain where the stress response is linear, ${\gamma < \gamma _\text {c}}$) for each system against **a** volume fraction ${\phi }$ and **b** attractive force ${F}_{\text {A}}$ for ${\phi }=0.620$. For **a** the dashed line indicates ${G~=~K|\phi ~-~\phi _{c}|}$. In **b** the blue dashed line indicates where the ${F}_{\text {A}}=0$ point would lie; this point is circled in blue in (**a**). **c**, **d** Plots of the viscosity (calculated as outlined in Sect. 2) ${\eta /\eta _{f}}$ for each system **c** in the non-attractive case (${F}_{\text {A}}=0$) as a function of volume fraction ${\phi }$ and **d** with varying attractive forces ${F}_{\text {A}}$ for the case where ${\phi }=0.620$. For **c** the dashed line indicates ${\eta /\eta _{f}~=~A|\phi ~-~\phi _{c}|^{-2}}$, which holds for all values of ${\phi }$ below ${\phi _{c}}$. **e** Presents a phase diagram showing which values of ${F}_{\text {A}}$ and ${\phi }$ exhibit jammed (green) or flowing (red) behaviour at small shear strains: certain intermediate cases are shaded white (colour figure online)

The consequences of this changing structure at the microscopic level are illustrated in Fig. 3. In Fig. 3a the dashed line indicates ${G~=~K|\phi ~-~\phi _{c}|}$, which holds for all values of ${\phi }$ above ${\phi _{c}}$. In this case, ${K}=2.7$ and ${\phi _{c}}=0.648$. In Fig. 3b the blue dashed line indicates where the ${F}_{\text {A}}=0$ point would lie; this point is circled in blue in Fig. 3a. In Fig. 3c the dashed line indicates ${\eta /\eta _{f}~=~A|\phi ~-~\phi _{c}|^{-2}}$, which holds for all values of ${\phi }$ below ${\phi _{c}}$. In this case, ${A}=0.08$ and ${\phi _{c}}=0.648$. In Fig. 3e we present a phase diagram showing which values of ${F}_{\text {A}}$ and ${\phi }$ exhibit jammed (green) or flowing (red) behaviour at small shear strains. From Fig. 3 we can see that increasing the attractive force has an effect which is comparable to that of increasing the volume fraction, thereby presenting an extra way in which we can tune the macroscopic properties of an assembly of particles. These two transitions are equivalent in the sense that they delineate comparable magnitudes of the shear modulus, with the transition occurring when $\sigma /\gamma =0.0001\rightarrow 0.001$ *k*/*d* in each case. They are, however, of different origin, with Fig. 3a representing solidification by percolation of *repulsive* interactions and Fig. 3b representing solidification by percolation of *attractive* interactions. The shear moduli clearly increase with volume fraction, which can be understood in terms of the increasing particle density causing the particles to be forced into one another to a greater extent - resulting in a higher degree of stress for a given applied strain value. Similarly with increasing attractive force (the data shown is for a volume fraction ${\phi }=0.620$), the particles are pulled towards each other more tightly and hence produce a larger elastic response upon shearing. In (c) and (d) it is clear that viscosity increases with volume fraction, which makes sense since the increasing particle density means that particles experience a greater resistance on average to any movement through the background liquid—with both contact and hydrodynamic contributions to this resistance. A larger ${F}_{\text {A}}$ will also increase the viscosity as the greater particle–particle attraction acts to impede motion through the system. Figure 3e shows the phase diagram for the results investigated here, and it illustrates how we can either move vertically and increase ${F}_{\text {A}}$, or move horizontally—increasing ${\phi }$—to achieve solid-like behaviour at small shear strain.

**Fig. 4:**
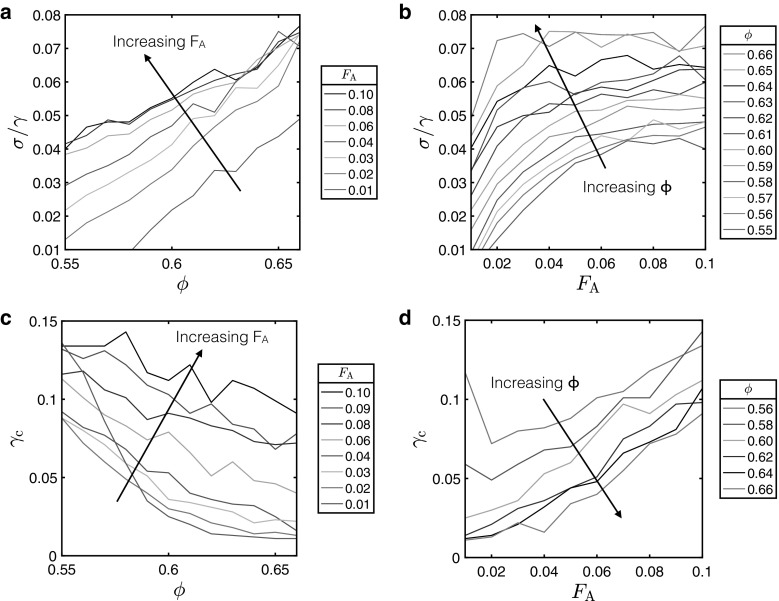
Variation of shear modulus and critical strain with volume fraction and attractive force. **a**, **b** How the shear modulus (${\sigma }$/${\gamma }$) changes with (**a**) volume fraction ${\phi }$ for a range of values of ${F}_{\text {A}}$, and **b** attractive force ${F}_{\text {A}}$ for a range of values of ${\phi }$. **c**, **d** Variation in critical strain (${\gamma _\text {c}}$) as a function of **c** volume fraction ${\phi }$ for a range of values of ${F}_{\text {A}}$, and **d** attractive force ${F}_{\text {A}}$ for a range of values of ${\phi }$. In each case the shear modulus was calculated by taking the gradient of the stress-strain plot between the origin and the critical strain value ${\gamma _\text {c}}$—the point at which the percentage of initial contacts remaining (see Fig.  2) drops below 99.85%—this was deemed to be the value which gave the best fit for all stress–strain plots

Finally we discuss how changing ${\phi }$ and ${F}_{\text {A}}$ affect both the shear modulus ${\sigma }$/${\gamma }$ and the critical strain $\gamma _c$ in the range ${F}_{\text {A}}=0.01\,\hbox {to}\,0.1$. $\gamma _c$ in each case was chosen as the shear strain value at which the percentage of initial contacts remaining (see Fig. 2) fell below 99.85%, as described above. This was found to give the best agreement with the simulation results as the point at which each system had yielded. The shear moduli $\sigma /\gamma$ values were then calculated by taking the initial gradient of each stress-strain plot up to $\gamma _c$. The plots in Fig. 4 depict the change in the shear modulus and critical strain with volume fraction and attractive force. From (a) it is clear that, as we saw in Fig. 3, $\sigma /\gamma$ increases with ${\varphi }$. This can be rationalised in terms of the increasing elastic response generated for a denser particle suspension. Similarly (b) shows again that with increasing ${F}_{\text {A}}$ the particles are bound together more tightly and hence produce a greater elastic response upon the application of shear. $\sigma /\gamma$ appears to reach a maximum value for each volume fraction, suggesting that even stronger attractive forces will do little to change the rheology of the materials. The critical strain generally decreases with increasing ${\phi }$, as can be seen in (c). This can be understood in terms of a greater particle density allowing for less elastic movement of particles before yielding. As might be expected a stronger attractive force between particles increases the critical strain, since the particles are more tightly bound, and as such can endure a higher shear strain before the system yields. The apparent decrease in $\gamma _c$ from $F_\text {A}=0.01 ~\hbox {to}~ 0.02$ for some of the lower values of $\phi$ appears because these less dense systems do not jam under shear, and hence deform like liquids rather than like solids.

## Conclusion

We present here the findings of a study into how isotropic attractions between suspended particles and precisely controlled volume fractions can give extra handles through which one may control the macroscopic flow properties of densely packed athermal suspensions. This is relevant both to the design of future yield stress materials with industrial and consumer product applications, but also to understanding the challenging processing and transportation of attractive, dense systems such as powers, pastes and wet grains. A liquid-like suspension with a volume fraction marginally below jamming can be made to deform in a solid-like way by means of a simple (and rather weak) attractive force acting between the particles. Similarly if the attractions between particles in a suspension can be suppressed, for example by specific tuning of interactions or by dehumidification of processing atmosphere, the material will behave as if its density has been decreased, allowing for a more uninhibited flow of material. These findings pave the way for more material-specific simulations involving, perhaps, tuneable, surface-mobile and site-specific interactions between particles which will further enhance our ability to predict and control the behaviour of soft matter systems.

## References

[1] Mezzenga R, Schurtenberger P, Burbidge A, Michel M (2005). Understanding foods as soft materials. Nat. Mater..

[2] Coussot P (2014). Yield stress fluid flows: a review of experimental data. J. Non Newton. Fluid Mech..

[3] Hornbaker DJ, Albert R, Albert I, Barabasi a L, Schiffer P (1997). What keeps sandcastles standing?. Nature.

[4] van Hecke M (2010). Jamming of soft particles: geometry, mechanics, scaling and isostaticity. J. Phys. Condens. Matter.

[5] Grob M, Heussinger C, Zippelius A (2014). Jamming of frictional particles: a nonequilibrium first-order phase transition. Phys. Rev. E.

[6] Gu Y, Chialvo S, Sundaresan S (2014). Rheology of cohesive granular materials across multiple dense-flow regimes. Phys. Rev. E.

[7] Landrum BJ, Russel WB, Zia RN (2016). Delayed yield in colloidal gels: creep, flow, and re-entrant solid regimes. J. Rheol..

[8] Beales PA, Vanderlick TK (2007). Specific binding of different vesicle populations by the hybridization of membrane-anchored DNA. J. Phys. Chem. A.

[9] Geerts N, Eiser E (2010). DNA-functionalized colloids: Physical properties and applications. Soft Matter.

[10] Song M, Ding Y, Snyder MA, Mittal J (2016). Effect of nonionic surfactant on association/dissociation transition of DNA-functionalized colloids. Langmuir.

[11] Van der Meulen SA, Helms G, Dogterom M (2015). Solid colloids with surface-mobile linkers. J. Phys. Condens. Matter.

[12] Pantaleev S, Yordanova S, Janda A, Marigo M, Ooi JY (2017). An experimentally validated dem study of powder mixing in a paddle blade mixer. Powder Technol..

[13] Thakur SC, Morrissey JP, Sun J, Chen J, Ooi JY (2014). Micromechanical analysis of cohesive granular materials using the discrete element method with an adhesive elasto-plastic contact model. Granular Matter.

[14] Morrissey, J.P.: Discrete element modelling of iron ore pellets to include the effects of moisture and fines. PhD thesis, University of Edinburgh (2013)

[15] Brown E, Forman NA, Orellana CS, Zhang H, Maynor BW, Betts DE, DeSimone JM, Jaeger HM (2010). Generality of shear thickening in dense suspensions. Nat. Mater..

[16] Irani E, Chaudhuri P, Heussinger C (2014). Impact of attractive interactions on the rheology of dense athermal particles. Phys. Rev. Lett..

[17] Zheng W, Liu H, Xu N (2016). Shear-induced solidification of athermal systems with weak attraction. Phys. Rev. E.

[18] Irani E, Chaudhuri P, Heussinger C (2016). Athermal rheology of weakly attractive soft particles. Phys. Rev. E.

[19] Chaudhuri P, Berthier L, Bocquet L (2012). Inhomogeneous shear flows in soft jammed materials with tunable attractive forces. Phys. Rev. E.

[20] Liu W, Jin Y, Chen S, Makse HA, Li S (2017). Equation of state for random sphere packings with arbitrary adhesion and friction. Soft Matter.

[21] Varga Z, Swan J (2016). Hydrodynamic interactions enhance gelation in dispersions of colloids with short-ranged attraction and long-ranged repulsion. Soft Matter.

[22] Singh A, Magnanimo V, Saitoh K, Luding S (2014). Effect of cohesion on shear banding in quasistatic granular materials. Phys. Rev. E.

[23] Singh, A., Magnanimo, V., Luding, S.: A contact model for sticking of adhesive meso-particles. arXiv preprint arXiv:1503.03720 (2015)

[24] Plimpton S (1995). Fast parallel algorithms for short range molecular dynamics. J. Comput. Phys..

[25] Lu PJ, Zaccarelli E, Ciulla F, Schofield AB, Sciortino F, Weitz DA (2008). Gelation of particles with short-range attraction. Nature.

[26] Peters F, Ghigliotti G, Gallier S, Blanc F, Lemaire E, Lobry L (2016). Rheology of non-brownian suspensions of rough frictional particles under shear reversal: a numerical study. J. Rheol..

[27] He H, Thorpe MF (1985). Elastic properties of glasses. Phys. Rev. Lett..

